# 1-Carb­oxy­methyl-3-octylimidazolium bromide

**DOI:** 10.1107/S1600536811022094

**Published:** 2011-06-18

**Authors:** Nassir N. Al-Mohammed, Yatimah Alias, Zanariah Abdullah, Hamid Khaledi

**Affiliations:** a Department of Chemistry, University of Malaya, 50603 Kuala Lumpur, Malaysia

## Abstract

In the title compound, C_13_H_23_N_2_O_2_^+^·Br^−^, the octyl chain has an all-*trans* conformation. In the crystal, the cations are linked by C—H⋯O bonds into a zigzag chain along the *b* axis. The bromide anions further link the chains *via* C—H⋯Br inter­actions into a two-dimensional array parallel to the *ab* plane. An O—H⋯Br interaction is also observed.

## Related literature

For related structures, see: Wei *et al.* (2009 ▶); Chen *et al.* (2009 ▶).
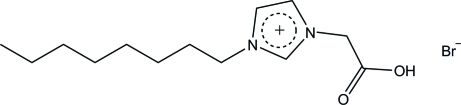


## Experimental

### 

#### Crystal data

C_13_H_23_N_2_O^2+^·Br^−^
*M* *_r_* = 319.24
Monoclinic, 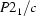

*a* = 7.6745 (2) Å
*b* = 4.6176 (1) Å
*c* = 41.8663 (9) Å
β = 92.167 (1)°
*V* = 1482.59 (6) Å^3^
*Z* = 4
Mo *K*α radiation
μ = 2.77 mm^−1^
*T* = 100 K
0.21 × 0.19 × 0.06 mm

#### Data collection

Bruker APEXII CCD diffractometer
Absorption correction: multi-scan (*SADABS*; Sheldrick, 1996 ▶) *T*_min_ = 0.594, *T*_max_ = 0.851
10905 measured reflections
2761 independent reflections
2678 reflections with *I* > 2σ(*I*)
*R*_int_ = 0.023

#### Refinement

*R*[*F* ^2^ > 2σ(*F* ^2^)] = 0.049
*wR*(*F* ^2^) = 0.095
*S* = 1.43
2761 reflections
167 parameters
1 restraint
H atoms treated by a mixture of independent and constrained refinement
Δρ_max_ = 1.25 e Å^−3^
Δρ_min_ = −2.19 e Å^−3^

### 

Data collection: *APEX2* (Bruker, 2007 ▶); cell refinement: *SAINT* (Bruker, 2007 ▶); data reduction: *SAINT*; program(s) used to solve structure: *SHELXS97* (Sheldrick, 2008 ▶); program(s) used to refine structure: *SHELXL97* (Sheldrick, 2008 ▶); molecular graphics: *X-SEED* (Barbour, 2001 ▶); software used to prepare material for publication: *SHELXL97* and *publCIF* (Westrip, 2010 ▶).

## Supplementary Material

Crystal structure: contains datablock(s) I, New_Global_Publ_Block. DOI: 10.1107/S1600536811022094/is2721sup1.cif

Structure factors: contains datablock(s) I. DOI: 10.1107/S1600536811022094/is2721Isup2.hkl

Additional supplementary materials:  crystallographic information; 3D view; checkCIF report

## Figures and Tables

**Table 1:** Hydrogen-bond geometry (Å, °)

*D*—H⋯*A*	*D*—H	H⋯*A*	*D*⋯*A*	*D*—H⋯*A*
C6—H6*B*⋯Br1^i^	0.99	2.89	3.772 (4)	148
C5—H5⋯Br1^ii^	0.95	2.91	3.681 (4)	139
C4—H4⋯O2^iii^	0.95	2.25	3.151 (5)	158
C3—H3⋯Br1^i^	0.95	2.82	3.593 (4)	139
C2—H2*B*⋯Br1^iv^	0.99	2.90	3.676 (4)	136
C2—H2*A*⋯O2^v^	0.99	2.44	3.328 (5)	150
O1—H1⋯Br1	0.84 (2)	2.33 (2)	3.153 (3)	168 (4)

Symmetry codes: (i) 
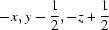
; (ii) 
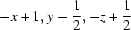
; (iii) 
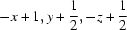
; (iv) 
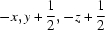
; (v) 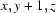.

**Fig. 1.**
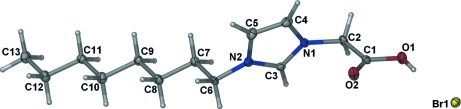
The molecular structure of the title compound with displacement ellipsoids drawn at 50% probability level. Hydrogen atoms are drawn as spheres of arbitrary radius.

**Fig. 2.**
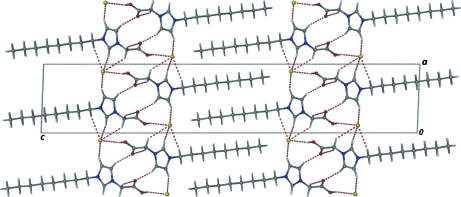
Packing view looking down the crystallographic b axis.

## supplementary crystallographic information

### Comment

The imidazolium ring is *N*-bound to a long alkyl chain, namely an octyl chain, and a carboxymethyl group. The alkyl chain adopts an all-*trans* conformation, as observed in similar structures (Wei *et al.*, 2009; Chen *et al.*, 2009). The carboxy group and the imidazolium ring subtend an N1—C2—C1 angle of 110.7 (3)°. In the crystal, the cationic moieties are bonded *via* C—H···O interactions (Table 1) into infinite chains along the *b* axis. The bromide anions link the cationic chains through C—H···Br interactions into layers parallel to the *ab* plane (Fig. 2).

### Experimental

A solution of bromoacetic acid (1 g, 7.2 mmol) and octylimidazole (1.29 g, 7.2 mmol) in dry 1,4-dioxane (10 ml) was stirred under nitrogen atmosphere at 70 °C for 7 hr. The solution was then poured into dichloromethane (25 ml) and extracted with distilled water (50 ml). The aqueous solution was evaporated under vacuum to give a viscous oil which crystallized from THF to yield the colorless crystals of the title compound.

### Refinement

The C-bound H atoms were placed at calculated positions [C—H distances of 0.95 (*Ar*), 0.98 (*methyl*) and 0.99 (*methylene*) Å] and refined as riding atoms, with *U*_iso_(H) set to 1.2(1.5 for methyl)*U*_eq_(C). The carboxylic hydrogen atom was located in a difference Fourier map and refind with a dinstance restraint of O—H 0.84 (2) Å. The maximum and minimum residual electron density peaks of 1.25 and -2.19 e Å^-3^, respectively, were located 2.06 and 1.85 Å from atom Br1.

### Crystal data

**Table:**

C_13_H_23_N_2_O^2+^·Br^−^	*F*(000) = 664
*M**_r_* = 319.24	*D*_x_ = 1.430 Mg m^−^^3^
Monoclinic, *P*2_1_/*c*	Mo *K*α radiation, λ = 0.71073 Å
Hall symbol: -P 2ybc	Cell parameters from 9093 reflections
*a* = 7.6745 (2) Å	θ = 2.7–30.4°
*b* = 4.6176 (1) Å	µ = 2.77 mm^−^^1^
*c* = 41.8663 (9) Å	*T* = 100 K
β = 92.167 (1)°	Plate, colorless
*V* = 1482.59 (6) Å^3^	0.21 × 0.19 × 0.06 mm
*Z* = 4	

### Data collection

**Table:**

Bruker APEXII CCD diffractometer	2761 independent reflections
Radiation source: fine-focus sealed tube	2678 reflections with *I* > 2σ(*I*)
graphite	*R*_int_ = 0.023
φ and ω scans	θ_max_ = 25.5°, θ_min_ = 2.7°
Absorption correction: multi-scan (*SADABS*; Sheldrick, 1996)	*h* = −9→9
*T*_min_ = 0.594, *T*_max_ = 0.851	*k* = −5→5
10905 measured reflections	*l* = −50→50

### Refinement

**Table:**

Refinement on *F*^2^	Primary atom site location: structure-invariant direct methods
Least-squares matrix: full	Secondary atom site location: difference Fourier map
*R*[*F*^2^ > 2σ(*F*^2^)] = 0.049	Hydrogen site location: inferred from neighbouring sites
*wR*(*F*^2^) = 0.095	H atoms treated by a mixture of independent and constrained refinement
*S* = 1.43	*w* = 1/[σ^2^(*F*_o_^2^) + (0.*P*)^2^ + 5.6415*P*] where *P* = (*F*_o_^2^ + 2*F*_c_^2^)/3
2761 reflections	(Δ/σ)_max_ < 0.001
167 parameters	Δρ_max_ = 1.25 e Å^−^^3^
1 restraint	Δρ_min_ = −2.19 e Å^−^^3^

### Special details

**Table:**

Geometry. All e.s.d.'s (except the e.s.d. in the dihedral angle between two l.s. planes) are estimated using the full covariance matrix. The cell e.s.d.'s are taken into account individually in the estimation of e.s.d.'s in distances, angles and torsion angles; correlations between e.s.d.'s in cell parameters are only used when they are defined by crystal symmetry. An approximate (isotropic) treatment of cell e.s.d.'s is used for estimating e.s.d.'s involving l.s. planes.
Refinement. Refinement of *F*^2^ against ALL reflections. The weighted *R*-factor *wR* and goodness of fit *S* are based on *F*^2^, conventional *R*-factors *R* are based on *F*, with *F* set to zero for negative *F*^2^. The threshold expression of *F*^2^ > σ(*F*^2^) is used only for calculating *R*-factors(gt) *etc*. and is not relevant to the choice of reflections for refinement. *R*-factors based on *F*^2^ are statistically about twice as large as those based on *F*, and *R*- factors based on ALL data will be even larger.

### Fractional atomic coordinates and isotropic or equivalent isotropic displacement parameters (Å^2^)

**Table:**

	*x*	*y*	*z*	*U*_iso_*/*U*_eq_	
Br1	0.10811 (5)	0.60448 (9)	0.158623 (9)	0.01633 (12)	
O1	0.1327 (4)	0.9207 (7)	0.22540 (7)	0.0206 (6)	
H1	0.140 (6)	0.823 (9)	0.2087 (7)	0.025*	
O2	0.3056 (4)	0.5633 (7)	0.24528 (7)	0.0223 (7)	
N1	0.3042 (4)	0.7879 (7)	0.30550 (7)	0.0135 (7)	
N2	0.3636 (4)	0.4977 (8)	0.34476 (7)	0.0141 (7)	
C1	0.2224 (5)	0.7809 (9)	0.24846 (9)	0.0145 (8)	
C2	0.2071 (6)	0.9390 (9)	0.27999 (9)	0.0179 (9)	
H2A	0.2530	1.1383	0.2780	0.021*	
H2B	0.0829	0.9524	0.2853	0.021*	
C3	0.2346 (5)	0.6121 (9)	0.32676 (9)	0.0144 (8)	
H3	0.1137	0.5747	0.3287	0.017*	
C4	0.4832 (5)	0.7869 (10)	0.31017 (10)	0.0191 (9)	
H4	0.5648	0.8927	0.2983	0.023*	
C5	0.5201 (5)	0.6074 (10)	0.33479 (9)	0.0191 (9)	
H5	0.6328	0.5640	0.3437	0.023*	
C6	0.3422 (5)	0.3118 (9)	0.37260 (9)	0.0168 (9)	
H6A	0.4475	0.1900	0.3759	0.020*	
H6B	0.2413	0.1816	0.3686	0.020*	
C7	0.3137 (5)	0.4881 (9)	0.40246 (9)	0.0163 (8)	
H7A	0.2055	0.6032	0.3994	0.020*	
H7B	0.4120	0.6248	0.4059	0.020*	
C8	0.2994 (5)	0.2982 (9)	0.43200 (9)	0.0172 (8)	
H8A	0.4069	0.1809	0.4348	0.021*	
H8B	0.2002	0.1632	0.4286	0.021*	
C9	0.2731 (5)	0.4719 (9)	0.46238 (9)	0.0158 (8)	
H9A	0.3717	0.6083	0.4656	0.019*	
H9B	0.1651	0.5879	0.4596	0.019*	
C10	0.2602 (6)	0.2852 (10)	0.49212 (10)	0.0195 (9)	
H10A	0.3684	0.1697	0.4949	0.023*	
H10B	0.1618	0.1484	0.4889	0.023*	
C11	0.2332 (5)	0.4592 (9)	0.52259 (9)	0.0162 (9)	
H11A	0.3315	0.5965	0.5258	0.019*	
H11B	0.1250	0.5744	0.5198	0.019*	
C12	0.2207 (6)	0.2733 (10)	0.55249 (9)	0.0196 (9)	
H12A	0.3290	0.1586	0.5554	0.023*	
H12B	0.1225	0.1359	0.5494	0.023*	
C13	0.1934 (6)	0.4506 (10)	0.58268 (9)	0.0215 (10)	
H13A	0.2926	0.5814	0.5864	0.032*	
H13B	0.1840	0.3199	0.6010	0.032*	
H13C	0.0860	0.5643	0.5800	0.032*	

### Atomic displacement parameters (Å^2^)

**Table:**

	*U*^11^	*U*^22^	*U*^33^	*U*^12^	*U*^13^	*U*^23^
Br1	0.0160 (2)	0.0183 (2)	0.01477 (19)	0.00006 (18)	0.00112 (13)	−0.00132 (18)
O1	0.0278 (15)	0.0187 (17)	0.0153 (14)	0.0039 (14)	0.0000 (12)	0.0001 (13)
O2	0.0315 (16)	0.0190 (18)	0.0164 (14)	0.0086 (14)	0.0024 (12)	−0.0021 (13)
N1	0.0177 (16)	0.0132 (17)	0.0098 (15)	−0.0009 (14)	0.0026 (13)	−0.0002 (13)
N2	0.0145 (16)	0.0162 (17)	0.0118 (16)	−0.0005 (14)	0.0018 (13)	0.0000 (13)
C1	0.0155 (19)	0.015 (2)	0.0131 (19)	−0.0032 (17)	0.0029 (15)	0.0017 (16)
C2	0.027 (2)	0.010 (2)	0.0162 (19)	0.0048 (18)	0.0005 (16)	0.0010 (16)
C3	0.0160 (18)	0.0128 (19)	0.0144 (18)	−0.0016 (17)	0.0004 (14)	−0.0021 (17)
C4	0.0145 (19)	0.025 (2)	0.018 (2)	−0.0043 (18)	0.0032 (16)	−0.0028 (18)
C5	0.0131 (18)	0.026 (2)	0.018 (2)	0.0021 (19)	0.0008 (15)	−0.0008 (19)
C6	0.021 (2)	0.010 (2)	0.019 (2)	−0.0004 (17)	−0.0022 (16)	0.0039 (16)
C7	0.0177 (19)	0.016 (2)	0.0150 (19)	0.0011 (17)	0.0002 (15)	0.0010 (16)
C8	0.0175 (19)	0.015 (2)	0.019 (2)	0.0016 (17)	0.0007 (16)	0.0014 (17)
C9	0.0159 (19)	0.015 (2)	0.0166 (19)	0.0021 (16)	−0.0006 (15)	0.0014 (16)
C10	0.020 (2)	0.020 (2)	0.019 (2)	0.0037 (18)	0.0011 (16)	−0.0003 (18)
C11	0.0158 (19)	0.014 (2)	0.019 (2)	0.0012 (16)	0.0013 (16)	0.0010 (17)
C12	0.021 (2)	0.021 (2)	0.017 (2)	0.0009 (18)	0.0031 (16)	0.0011 (18)
C13	0.025 (2)	0.024 (3)	0.016 (2)	0.0012 (19)	0.0033 (16)	−0.0001 (18)

### Geometric parameters (Å, °)

**Table:**

O1—C1	1.332 (5)	C7—H7A	0.9900
O1—H1	0.837 (19)	C7—H7B	0.9900
O2—C1	1.200 (5)	C8—C9	1.524 (5)
N1—C3	1.331 (5)	C8—H8A	0.9900
N1—C4	1.381 (5)	C8—H8B	0.9900
N1—C2	1.457 (5)	C9—C10	1.521 (6)
N2—C3	1.331 (5)	C9—H9A	0.9900
N2—C5	1.382 (5)	C9—H9B	0.9900
N2—C6	1.462 (5)	C10—C11	1.528 (6)
C1—C2	1.517 (5)	C10—H10A	0.9900
C2—H2A	0.9900	C10—H10B	0.9900
C2—H2B	0.9900	C11—C12	1.524 (6)
C3—H3	0.9500	C11—H11A	0.9900
C4—C5	1.344 (6)	C11—H11B	0.9900
C4—H4	0.9500	C12—C13	1.527 (6)
C5—H5	0.9500	C12—H12A	0.9900
C6—C7	1.515 (6)	C12—H12B	0.9900
C6—H6A	0.9900	C13—H13A	0.9800
C6—H6B	0.9900	C13—H13B	0.9800
C7—C8	1.523 (5)	C13—H13C	0.9800
			
C1—O1—H1	107 (3)	C7—C8—C9	113.0 (3)
C3—N1—C4	109.0 (3)	C7—C8—H8A	109.0
C3—N1—C2	125.1 (3)	C9—C8—H8A	109.0
C4—N1—C2	125.7 (3)	C7—C8—H8B	109.0
C3—N2—C5	108.6 (3)	C9—C8—H8B	109.0
C3—N2—C6	125.5 (3)	H8A—C8—H8B	107.8
C5—N2—C6	125.5 (3)	C10—C9—C8	113.6 (3)
O2—C1—O1	126.0 (4)	C10—C9—H9A	108.9
O2—C1—C2	123.9 (4)	C8—C9—H9A	108.9
O1—C1—C2	110.0 (3)	C10—C9—H9B	108.9
N1—C2—C1	110.7 (3)	C8—C9—H9B	108.9
N1—C2—H2A	109.5	H9A—C9—H9B	107.7
C1—C2—H2A	109.5	C9—C10—C11	113.6 (4)
N1—C2—H2B	109.5	C9—C10—H10A	108.8
C1—C2—H2B	109.5	C11—C10—H10A	108.8
H2A—C2—H2B	108.1	C9—C10—H10B	108.8
N2—C3—N1	108.2 (3)	C11—C10—H10B	108.8
N2—C3—H3	125.9	H10A—C10—H10B	107.7
N1—C3—H3	125.9	C12—C11—C10	113.9 (3)
C5—C4—N1	106.9 (4)	C12—C11—H11A	108.8
C5—C4—H4	126.6	C10—C11—H11A	108.8
N1—C4—H4	126.6	C12—C11—H11B	108.8
C4—C5—N2	107.3 (3)	C10—C11—H11B	108.8
C4—C5—H5	126.3	H11A—C11—H11B	107.7
N2—C5—H5	126.3	C11—C12—C13	113.1 (4)
N2—C6—C7	111.5 (3)	C11—C12—H12A	109.0
N2—C6—H6A	109.3	C13—C12—H12A	109.0
C7—C6—H6A	109.3	C11—C12—H12B	109.0
N2—C6—H6B	109.3	C13—C12—H12B	109.0
C7—C6—H6B	109.3	H12A—C12—H12B	107.8
H6A—C6—H6B	108.0	C12—C13—H13A	109.5
C6—C7—C8	112.2 (3)	C12—C13—H13B	109.5
C6—C7—H7A	109.2	H13A—C13—H13B	109.5
C8—C7—H7A	109.2	C12—C13—H13C	109.5
C6—C7—H7B	109.2	H13A—C13—H13C	109.5
C8—C7—H7B	109.2	H13B—C13—H13C	109.5
H7A—C7—H7B	107.9		

### Hydrogen-bond geometry (Å, °)

**Table:**

*D*—H···*A*	*D*—H	H···*A*	*D*···*A*	*D*—H···*A*
C6—H6B···Br1^i^	0.99	2.89	3.772 (4)	148
C5—H5···Br1^ii^	0.95	2.91	3.681 (4)	139
C4—H4···O2^iii^	0.95	2.25	3.151 (5)	158
C3—H3···Br1^i^	0.95	2.82	3.593 (4)	139
C2—H2B···Br1^iv^	0.99	2.90	3.676 (4)	136
C2—H2A···O2^v^	0.99	2.44	3.328 (5)	150
O1—H1···Br1	0.84 (2)	2.33 (2)	3.153 (3)	168 (4)

Symmetry codes: (i) −*x*, *y*−1/2, −*z*+1/2; (ii) −*x*+1, *y*−1/2, −*z*+1/2; (iii) −*x*+1, *y*+1/2, −*z*+1/2; (iv) −*x*, *y*+1/2, −*z*+1/2; (v) *x*, *y*+1, *z*.
